# Genomic Characterization of Local Croatian Sheep Breeds-Effective Population Size, Inbreeding & Signatures of Selection

**DOI:** 10.3390/ani14131928

**Published:** 2024-06-29

**Authors:** Jelena Ramljak, Marija Špehar, Dora Ceranac, Valentino Držaić, Ivan Pocrnić, Dolores Barać, Boro Mioč, Ivan Širić, Zdravko Barać, Ante Ivanković, Ante Kasap

**Affiliations:** 1 Faculty of Agriculture, University of Zagreb, 10000 Zagreb, Croatia; vdrzaic@agr.hr (V.D.); bmioc@agr.hr (B.M.); isiric@agr.hr (I.Š.); aivankovic@agr.hr (A.I.); akasap@agr.hr (A.K.); 2 Croatian Agency for Agriculture and Food, 10000 Zagreb, Croatia; marija.spehar@hapih.hr (M.Š.); dora.ceranac@hapih.hr (D.C.); dolores.barac@hapih.hr (D.B.); 3 The Roslin Institute, University of Edinburgh, Easter Bush Campus, Midlothian EH25 9RG, UK; ivan.pocrnic@roslin.ed.ac.uk; 4 Ministry of Agriculture, 10000 Zagreb, Croatia; zdravko.barac@mps.hr

**Keywords:** effective population size, genomic inbreeding, ROH island, OCS

## Abstract

**Simple Summary:**

The Istrian sheep (IS) and the Pag sheep (PS) are local Croatian breeds known for their delicious cheese and lamb meat. In order to develop an effective selection program in accordance with the principles of Optimum Contribution Selection (OCS), we estimated the linkage disequilibrium effective population size and conducted a comprehensive analysis of runs of homozygosity (ROH) on a substantial number of genotyped animals. In both breeds, there was strong evidence of recent inbreeding, but the estimate of genomic inbreeding (F_ROH_) in IS was twice as high as in PS and was on the edge of acceptable levels, so optimisation of mating plans is needed in the future to maintain the genetic variability. Signatures of selection were found only in IS and were associated with growth, feed intake, milk production, and immunity traits.

**Abstract:**

The Istrian (IS) and the Pag sheep (PS) are local Croatian breeds which provide significant income for the regional economy and have a cultural and traditional importance for the inhabitants. The aim of this study was to estimate some important population specific genetic parameters in IS (N = 1293) and PS (N = 2637) based on genome wide SNPs. Estimates of linkage disequilibrium effective population size (N_e_) evidenced more genetic variability in PS (N_e_ = 838) compared to IS (N_e_ = 197), regardless of historical time (both recent and ancient genetic variability). The discrepancy in the recent genetic variability between these breeds was additionally confirmed by the estimates of genomic inbreeding (F_ROH_), which was estimated to be notably higher in IS (F_ROH>2_ = 0.062) than in PS (F_ROH>2_ = 0.029). The average F_ROH2–4_, F_ROH4–8_, F_ROH8–16_, and F_ROH>16_ were 0.26, 1.65, 2.14, and 3.72 for IS and 0.22, 0.61, 0.75, and 1.58 for PS, thus evidencing a high contribution of recent inbreeding in the overall inbreeding. One ROH island with > 30% of SNP incidence in ROHs was detected in IS (OAR6; 34,253,440–38,238,124 bp) while there was no ROH islands detected in PS. Seven genes (CCSER1, HERC3, LCORL, NAP1L5, PKD2, PYURF, and SPP1) involved in growth, feed intake, milk production, immune responses, and resistance were associated with the found autozygosity. The results of this study represent the first comprehensive insight into genomic variability of these two Croatian local sheep breeds and will serve as a baseline for setting up the most promising strategy of genomic Optimum Contribution Selection.

## 1. Introduction

Public awareness of the need to preserve animal genetic resources due to their adaptation to environmental challenges and sustainable production [1,2] has become a burning issue in the last 20 years. This is particularly important when it comes to local breeds whose populations have been declining drastically (i.e., the original gene pools are being threatened), and many of them are already facing extinction. The decline in population size of many local breeds is mainly connected with their low productivity compared to some foreign purposely selected breeds (for meat, milk, or wool) [3,4]. To partially overcome this problem, local breeds have been historically crossed with breeds of high commercial value, but at the cost of losing their unique genetic profile. Also, in the dairy orientated sheep populations, crossbreeding failed to provide long term effects and they were abandoned in favor of within-population selection.

**Figure 1:**
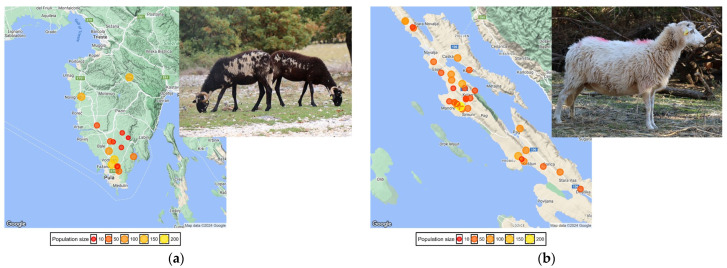
(**a**) Map of the Istrian Peninsula and Istrian sheep; (**b**) the Island of Pag and Pag sheep. Size and color of the circles are proportional to the number of sampled animals per flock.

Istrian sheep (IS) and Pag sheep (PS) are two Croatian local breeds originating from the Istrian Peninsula and the Island of Pag, respectively (Figure 1). The formation of the IS began around 1770 and the PS around 1870 by crossing local ewes with rams of foreign more productive breeds [5]. Nowadays, according to their production capacity, both IS and PS, are classified as a dairy orientated dual-purpose breeds (meat-milk). The registered population size of the IS is 2900 (1002 under selection) and the PS 30,000 (3627 under selection), respectively [6]. According to official data of Annual Report for year 2023 [4] obtained from milk recording based on the ICAR guidelines (Section 16, [5]), in the lactation length of approximately 5–6 months, the IS produces ~ 140 kg of milk with 6.3% milk fat and 5.6% protein and the PS produces ~120 kg of milk with 7.3% milk fat and 5.8% of protein. All the milk is used to produce traditional hard-full-fat cheese and curd cheese (albumin cheese made from whey). The products from these breeds are sold at high prices, predominately on the local market; and some of them, i.e., Pag cheese and Pag lamb meat have been labeled with the European quality marks (protected designation of origin (PDO).

Despite the fact that they are less productive compared to foreign highly selected breeds, local breeds are still of enormous importance today, not only as biological and cultural heritage (living monuments of history), but also as an irreplaceable genetic reservoir for future unpredictable environmental conditions and challenges. The preservation of genetic diversity is crucial for the successful survival of species in unpredictable climatic changes and epidemic outbreaks. The most efficient strategy is to keep local breeds alive, i.e., to preserve them in vivo in situ, while the conservation of genetic material stored in gene banks (conservation in vitro) should serve only as an additional precautionary measure [7]. In order to make them at least somewhat competitive with regard to foreign breeds, a long-term genetic gain through selection needs to be provided. However, selection focused solely on higher productivity inevitably poses a risk for preserving genetic variability. Therefore, it is crucial to balance between selection pressure and loss of genetic variability by mating animals with the highest breeding values while minimizing their mutual coancestry [8]. This strategy is called Optimum Contribution Selection (OCS), and nowadays, with the availability of whole-genome markers such as SNPs, the OCS can be implemented under the framework of genomic selection [9].

Prior to transition to any novel selection strategy, and especially the one that requires additional financial investments, it is wise to examine some important population genetic parameters that reflect genetic diversity within the population of interest (e.g., effective population size, inbreeding rate, genetic connectedness between the flocks, linkage disequilibrium, etc.). It is important not only to have some clues about the current state of the population under consideration but also to set up the most promising strategy for OCS. Linkage disequilibrium (LD), which represents the quantification of non-random association between markers in the population and runs of homozygosity (ROH) that represents the long homozygous stretches of DNA made of haplotypes being identical by descent (IBD), are one of the most important tools used to determine the above mentioned population genetic parameters in numerous recent conservation genetic studies [10,11]. In that sense, the aim of this study is to fill the gap in the knowledge of these important population-specific genetic parameters on the substantial number of genotyped animals and set up a baseline for the implementation of genomic OCS that will ensure competitiveness and long-term viability for Istrian and Pag sheep breeds.

## 2. Materials and Methods

### 2.1. Sampling and SNP Genotyping

Biological samples (ear tissue) were collected in a tissue sample unit (Allflex) and stored in a freezer until shipment to an accredited laboratory for genotyping (Weatherbys Scientific, Ireland). Sampling was carried out between 2019 and 2023 in 15 IS and 32 PS flocks (Figure 1, Table S1). All flocks in the study have been included in the breeding activities defined by the national selection program [12], which means that all sampled animals had known pedigree and available phenotypic information (dairy traits recorded following the ICAR guidelines [13]) and other descriptive information such as age, parity, type of birth, etc. All this information had to be known because the ultimate goal of genotyping was to initiate and set up long-term genomic OCS in these populations. Due to limited financial resources for genotyping and inability to genotype all the animals in these populations, we tried to capture most of the present genetic variability in the flocks by selecting the animals based on previous comprehensive analysis. Finally, a total of 1293 IS and 2637 PS were genotyped and included in the analysis.

Genotyping was performed with the OvineSNP50 BeadChip (Illumina, San Diego, CA, USA). Datasets obtained from different batches were used in the analysis. Each dataset was filtered using a confidence score of the genotyping (GC) that was set to 0.7. Markers placed on X and Y chromosome and markers with unknown position were removed from the analysis, and only autosomal SNPs located in unique positions were considered. Common SNPs across all datasets were identified, and the quality control of SNP marker data was performed using PLINK 1.9 [14]. Map files were updated to a reference map file using Illumina Infinium Ovine SNP50 v1 BeadChip (54,241 SNPs) (https://webserver.ibba.cnr.it/SNPchimp/index.php/download, accessed on 11 April 2024) to ensure that SNP names and locations were consistent across populations. The animal call rate, and SNP call rate were set to 0.9 and 0.95, respectively. Finally, a total of 33,965 SNPs and 1047 animals from IS and 2323 animals from PS were retained in analysis.

The effective population size (N_e_) based on LD was estimated separately for each breed using the GONE software [15]. Since the maximum sample size that GONE can handle is 1800 individuals, a representative sampling procedure was carried out using the R package BITEV2.1.0 [16] to calculate the N_e_ of the PS. For each population, 100 independent replicates were performed with the default settings. Genomic (di)similarity between the individuals of IS and PS breeds was estimated using multidimensional scaling (MDS) analysis in Plink v.1.9 [14].

### 2.2. Runs of Homozygosity

Detection of ROH was performed using PLINK 1.9 [14]. For the purpose of the ROH analysis, filtering the dataset on minor allele frequency (MAF), Hardy-Weinberg equilibrium (HWE), and linkage disequilibrium (LD) were not performed in order to avoid underestimation of the genome coverage with ROH segments [17]. As ROH detection is greatly influenced by a set of predefined parameters [17], we have customized them based on our dataset to provide a more robust and reliable analysis. Calling parameters used to identify ROH were: (i) a minimum length of 1 Mb was chosen to detect ROH, (ii) the maximum gap between consecutive SNPs within an ROH was set to 1 Mb, and (iii) the minimum density of SNPs in a genome window was one SNP every 150 Kb. The number of missing and heterozygous SNPs was adjusted for the ROH length category of 50K BeadChip according to Ferenčaković et al. [18] as follows: no missing and no heterozygous SNPs for categories up to 4 Mb, 1 missing and no heterozygous SNPs for categories of 4–8 Mb, 2 missing and no heterozygous SNPs for categories of 8–16 Mb and 4 missing and 1 heterozygous SNPs for categories >16 Mb. A separate ROH analysis was performed for each group of parameters. All defined parameters were kept the same for both breeds, and the only difference was in the minimum number of SNPs allowed in both the sliding window and final ROH segment, which was set to 50 for IS and 48 for PS.

The minimum number of SNPs in the sliding window and in the final ROH segment length was calculated using formula of Lencz et al. [19] as adapted by Purfield et al. [20]:

$$L=\frac{log_{e}\frac{\alpha }{n_{s}n_{i}}}{log_{e}\left(1-het\right)}$$

where *L* is the length of sliding window/final ROH segment, n_s_ is the number of SNPs per individual, *n_i_* is the number of animals, *het* is the mean heterozygosity across all SNPs, and *α* is the chosen significance level for type I errors.

The calculation of the threshold value for checking whether a SNP is designated as a part of an ROH was based on the formula by Meyermans et al. [17]:

$$t=\mathrm{floor}\;(\frac{N_{out}+1}{L},\;3)$$

where the threshold is *t*, *N_out_* is the desired number of SNPs on the outer sides of ROH segment that should not be included in ROH and *L* as same as in the formula above. *N_out_* was set to 2, and *t* was calculated to be 0.06.

Identified ROH segments were grouped into four classes according to their physical length: 2–4 Mb, 4–8 Mb, 8–16 Mb, and > 16 Mb, as typically done in similar studies [18,21].

### 2.3. Estimation of GenomicCoefficients of Inbreeding and Analysis of Signatures of Selection

Two different coefficients of genomic inbreeding were estimated: F_HOM_ and F_ROH_. The F_HOM_ was based on the difference between the observed and expected number of homozygous genotypes as follows:

$$F_{\mathrm{HOM}}=\frac{O_{HOM}-E_{HOM}}{L-E_{HOM}}$$

where *O_HOM_* was the number of observed homozygous genotypes, *E_HOM_* was the number of expected homozygous genotypes, and *L* was the total number of genotyped autosomal SNPs.

The F_ROH_ was based on the length of the total genome covered by ROH segments as follows:

$$F_{\mathrm{ROH}}=\frac{\sum L_{ROH}}{L_{auto}}$$

where $\sum L_{ROH}$ was the sum of all lengths of ROH detected and *L_auto_* was the length of autosome covered by SNPs (2.6 Mb).

Four different classes of F_ROH_ were calculated according to the previously determined four ROH length classes and overall, F_ROH_ > 2 Mb. Pearson’s correlation coefficient was used to compare the F_HOM_ and F_ROH>2_ inbreeding coefficients.

Detection of ROH islands was conducted according to Gorssen et al. [22], https://doi.org/10.17605/OSF.IO/XJTKV, accessed on 22 September 2023) for each breed separately. First, the ROH incidence was calculated according to Purfield et al. [23]:

$$ROH_{incidence\_snp}=\frac{N\;individuals\;with\;specific\;SNP\;in\;ROH}{Total\;N\;of\;genotyped\;individuals}$$

The adjacent SNP with the highest frequency of *ROH_incidence_snp_* was kept for scanning SNP window of 1 Mb. Then, the population-specific threshold was calculated according to *z*-scores from the distribution of ROH incidences and the top 0.1% of SNPs observed in ROH (*p*-value ≥ 0.999) were selected. In addition, a ROH had to be detected in at least 30% of the population to be declared as ROH island. Adjacent SNPs above the established threshold were merged and constituted the ROH islands [16,17]. The identified SNPs present in ROH island were used as input for the R package GALLO to find the mapped genes and QTLs within 1 Mb window-interval, as suggested by Fonseca et al. [24]. The gene database from Ensembl (https://ftp.ensembl.org/pub/release-111/gtf/ovis_aries_rambouillet/, accessed on 22 February 2024) was the basis for gene annotation. For QTL annotation, we used the QTL database from Animal QTLdb (https://www.animalgenome.org/cgi-bin/QTLdb/OA/index, accessed on 22 February 2024) and new ovine assembly QTLdb release52_sheepOAR4. To evaluate the functions of the selected genes, we conducted enrichment analyses using information from gene ontology (GO) and Kyoto Encyclopedia of Genes and Genomes (KEGG). Biological pathways associated with identified genes were obtained using the DAVID software v2024q1 [25,26] (https://david.ncifcrf.gov/; accessed on 15 May 2024).

## 3. Results

### 3.1. Genetic Relationship and Effective Population Size

**Figure 2:**
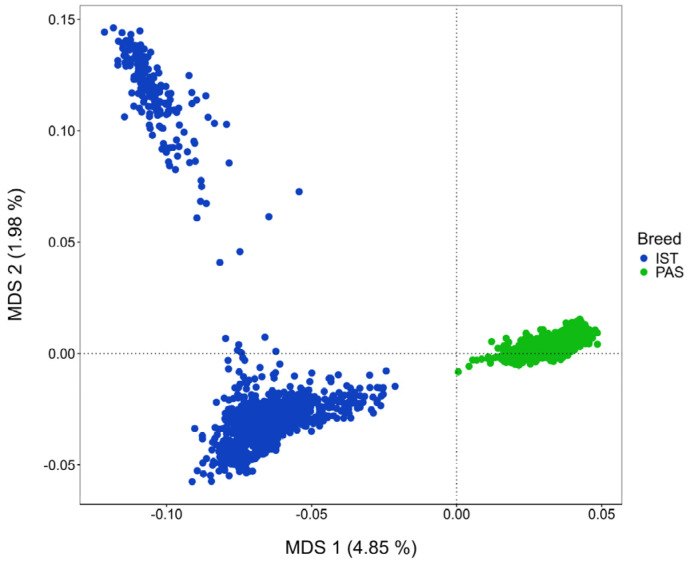
Genetic relationship defined with multidimensional scaling (MDS) analysis between the Istrian sheep (IST) and Pag sheep (PAS).

Genomic similarity between individuals belonging to IS and PS based on MDS is presented in Figure 2. The first two MDS components explained together approximately 7% of the total genomic variation, showing a clear distinction between the breeds (based on the first MDS component), but also, a clear subdivision of the IS into two genetically dissimilar subpopulations (based on the second MDS component).

**Figure 3:**
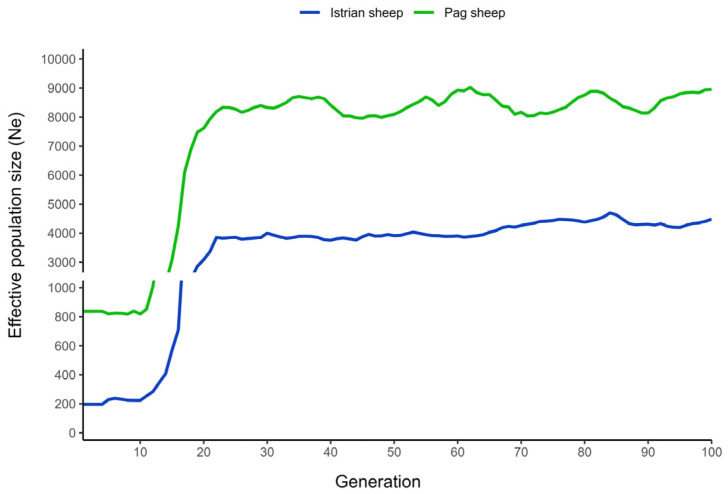
The effective population size (N_e_) estimates obtained by GONE software over the generations for Istrian and Pag sheep.

The estimation of the recent and historical N_e_ of IS and PS is presented in Figure 3. The recent N_e_ of PS and IS was estimated to be 838 and 197 animals, respectively. The historical N_e_ was traced back (estimated) up to 100 generations to provide a sufficient level of reliability, as suggested by Santiago et al. [15]. Despite the fact that both, the recent and historical N_e_ of PS, had been considerably higher than those of IS, a great similarity in the pattern of change in N_e_ over time was observed between these two breeds (Figure 3, parallelism of the estimated lines). Until approximately 16–18 generations ago, when the steep decline in N_e_ occurred, the N_e_ was estimated to be stable in both breeds and accounted for approximately 7.5 K animals in PS (the highest N_e_ = 7615) and 3 K animals in the IS (the highest N_e_ = 3084).

### 3.2. Genomic Inbreeding

The estimates of the coefficients of genomic inbreeding (F_HOM_ and F_ROH_ calculated for different classes of ROH lengths) are reported in Table 1. The average F_HOM_ in the IS and the PS was estimated to be 0.058 (min = −0.084, max = 0.482) and 0.039 (min = −0.070, max = 0.390), respectively. The average F_ROH>2_ for IS was 0.062 (from 0.001 to 0.439) and for PS 0.029 (from 0.001 to 0.342). Pearson correlations estimated between F_HOM_ and F_ROH>2_ in both breeds were high (>0.97). The average values for F_ROH2-4_, F_ROH4-8_, F_ROH8-16_, and F_ROH>16_ were 0.26, 1.65, 2.14 and 3.72 for IS and 0.22, 0.61, 0.75 and 1.58 for PS, respectively. The IS population showed a higher level of inbreeding than the PS, for each class of inbreeding coefficient except for F_ROH2-4_. The estimated F_ROH_ from segments above 4 Mb were approximately 2.5-fold higher in the IS, evidencing more of the recent inbreeding in the population of IS compared to PS.

The three most autozygous animals in the IS population had ROH genome coverage of 1068.89 Mb, 1047.89 Mb, and 984.52 Mb of the total autosomal genome resulting in the F_ROH_ of 0.406, 0.398, and 0.374, respectively. In the PS population, which had pretty much the same genome coverage with the ROH segments as the IS, only two individuals had a F_ROH_ > 0.300 (genome coverage with ROHs of 802.57 Mb and 782.49 Mb and F_ROH_ 0.317 and 0.305, respectively).

### 3.3. Characterisation of Runs of Homozygosity and Runs of Homozygosity Island

In IS and PS proportion of genome coverage, i.e., maximal detectable ROH length over the length of the autosomes was almost identical at 94% as indicator of the validity of the ROH analysis [17]. Mean ROH autosomal coverage per animal ranged from 2.7 to 28.4 Mb in IS and from 2.5 to 54.4 Mb in PS. The average number of ROH segments per animal was smaller in PS (5.94) compared to IS (13.05) (Table 1), and the average length of ROH (*L_ROH_*) per animal was 70.31 Kb for IS and 152.05 Kb for PS.

**Figure 4:**
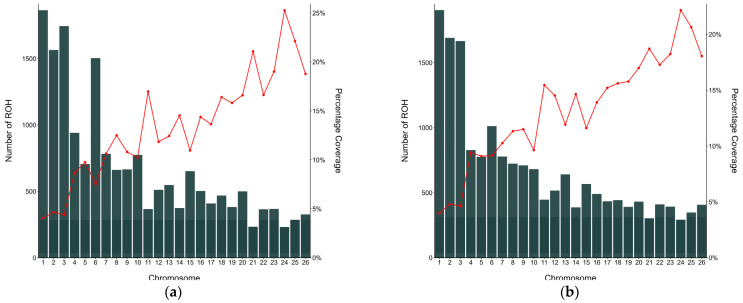
The distribution of detected ROH segments per chromosome (bars) and the average percentage of each chromosome covered by ROH (red line) for (**a**) Istrian sheep and (**b**) Pag sheep.

The number of ROHs per chromosome and the proportion of the chromosome covered by ROH in IS and PS are shown in Figure 4. The results indicate that ROH were unevenly distributed, with a descending tendency towards smaller chromosomes in terms of size. The highest number of ROH segments was found on chromosomes OAR1, OAR2, OAR3 (>1500 per chromosome), accounting for 37% and 30% of all ROHs in IS and PS, respectively. In both breeds, the number of ROHs on chromosome 6 was unexpectedly high (1187 in IS and 941 in PS), indicating the higher level of autozygosity on OAR6. The chromosomes with the least number of ROHs for both populations were OAR21 (212 in IS and 262 in PS) and OAR24 (198 in IS and 259 in PS), respectively.

The proportions of ROH of different class lengths for the two breeds are shown in Table 1. The distribution of ROHs according to their size showed that the majority of the detected segments were 4–8 Mb in both breeds; 43% in IS and 39% in PS. The least frequent ROH segments in IS were the short ones (ROH_2–4_; 15%), and in PS the long ones (ROH_>16_; 18%). Only 5 animals had very long segments ROH_>20_, and all of them belonged to the IS.

The determined average genomic inbreeding (F_ROH>2_) of IS was 6.24% and it was higher compared to the PS (2.88%) (Table 1). The partial coefficients of inbreeding based on different lengths of ROHs, as well as their portion in the overall inbreeding, increased with the lengths of ROHs. Accordingly, the highest level of inbreeding were F_ROH>16_ = 3.72 in IS and F_ROH>16_ = 1.58 in PS. The results are evidence of recent inbreeding in both populations.

### 3.4. Gene and QTL Annotation

**Figure 5:**
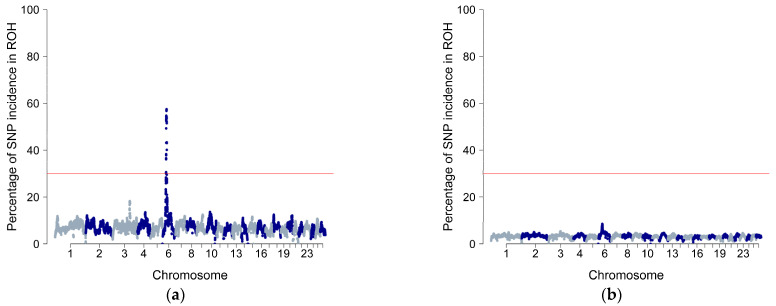
Runs of homozygosity islands in: (**a**) Istrian sheep (N = 1047) and (**b**) Pag sheep (N = 2323). The horizontal line corresponds to the threshold calculated as SNPs with a *p*-value for ROH incidence higher than 0.999.

The proportion of SNPs present in ROH was determined based on their frequency across individuals and ROH islands were defined as the regions with the top 0.999 SNPs of the percentile distribution. Figure 5 illustrates the frequency of SNPs within a ROH across autosomes, revealing homozygosity-rich genomic regions in both breeds.

In the IS population one ROH island with ~60% of SNP incidence in ROH was detected. ROH island was located on OAR6 chromosome and had a length of 3,984,684 bp (34,253,440–38,238,124 bp). Annotation analysis of genes and QTL was performed in 500-Kb regions upstream and downstream of the detected SNPs, with a significance threshold of 0.999. Seven genes (CCSER1, HERC3, LCORL, NAP1L5, PKD2, PYURF, and SPP1) were identified within the ROH islands and were classified as protein-coding in the Ensembl database (Table 2). Group of genes CCSER1 and PKD2 [27], LCORL and SPP1 [28], and PYURF are related to developmental biological pathways especially growth traits, feed intake, and milk production. In addition, HERC3 gene is associated with immune responses and resistance [29]. In the PS population, ROH incidence levels were low (up to 8%) without ROH islands detected (Figure 5b).

**Figure 6:**
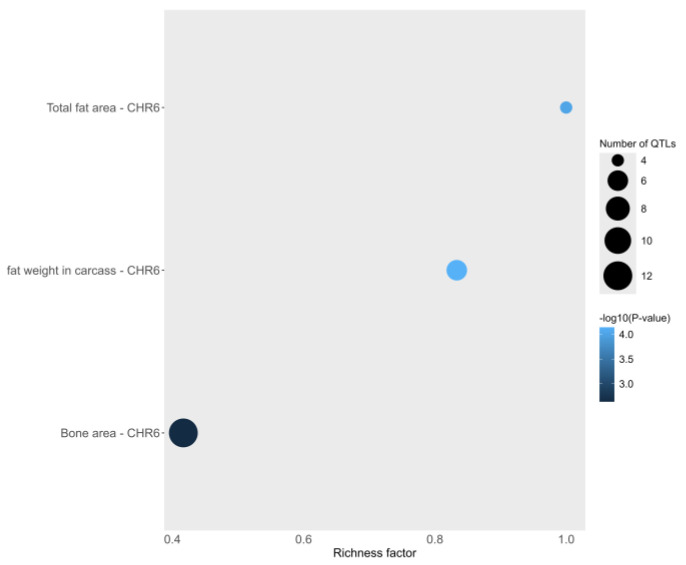
Bubble plots displaying top three traits identified in QTL enrichment analysis. The size of bubbles represents the number QTL observed for that trait. Shade of the blue represents the *p*-value scale (a darker color indicates smaller *p*-value). Richness factor is indicated on x-axis (it represents the ratio between the number of associated QTLs for a trait and the total number of QTLs in the data base for that trait).

Annotated QTL information was used to estimate the proportion of the QTL type for the respective QTL traits. A total of 19 QTLs related to important economic traits such as Meat and carcass traits (63%) and Production (38%) were identified in the IS. The most represented traits were “bone area”, “fat weight in carcass” and “total fat and dressing percentage” for Meat and carcass QTL as well as “body weight” and “total bone trait” for Production QTL (Table S2). Enrichment analysis was then performed for each annotated trait, and 11 enriched QTLs (out of 19) were meat and carcass-related QTLs (adjusted *p*-value < 0.05) (Figure 6). Several genes from this study were significantly enriched in some GO terms (PKD2, SPP1, PIGY) and KEGG pathways (SPP1, HERC3), which contributed to the identification and understanding of the biological processes and pathways of the aforementioned genes (Table S3). More specifically, genes were related to biological processes such as response to external stimulus (GO:0009605), regulation of homeostatic processes (GO:0042592), and regulation of biological quality (GO:0065008). In addition, genes were significantly enriched in the KEGG pathways, i.e., signaling pathways (SPP1; oas04620, oas04371), ECM-receptor interaction (SPP1; oas04512) and ubiquitin mediated proteolysis (HERC3; oas04120).

## 4. Discussion

### 4.1. Effective Population Size, Runs of Homozygosity and Genomic Inbreeding

The availability of SNP markers in recent decades has enabled more accurate study of many population-specific genetic parameters such as inbreeding, effective population size, genetic connectedness between flocks, etc. Since the estimates of these parameters based on genomic data were proven more accurate than their pedigree-based counterparts, the inclusion of genomic data in livestock breeding programs has increased rapidly over the last decade. Genomics has penetrated not only in breeding programs oriented towards the genetic progress of economically important traits but also in those seeking to preserve genetic variability. In Croatia, there has been an initiative to provide both of the above goals in two dairy-orientated local breeds (IS and PS) by implementing the basic principles of OCS in the flocks included in the national selection program. Therefore, to be able to set up the most promising OCS strategy and to be able to monitor selection goals, we decided to estimate some of the important population-specific parameters in these breeds.

A clear separation of breeds based on their genomic profile (Figure 2) was expected and logical, but the subdivision of the IS population was far from our expectations. In order to get some answers of this issue we investigated a potential contribution of a foreign genome in the pedigree, but found no such information. We are currently working on obtaining genotypes of breeds that historically contributed to the formation of IS and those information will be used for further structure analysis.

The estimates of the recent N_e_ differed substantially between IS and PS in favor of PS (838 vs. 197), but the ratio of N_e_ between the breeds was to huge extent proportional to the ratio of the census population size (N_c_). At the moment of analysis, this ratio was approximately 4:1 [6]. Tracing back the historical N_e_, a severe reduction in the both breeds started approximately 18 generations ago, coinciding with the period of 2nd World War and the post-war period. The time estimates were obtained by multiplying the number of generations by the average generation interval, which was estimated to be 3.8 in IS [30] and 4.4 in PS [31]. The war conditions, but also the change in the main production goal (transition from wool to milk) caused a severe reduction in N_c_ which was also reflected on the N_e_. To support of the claims regarding the historical decline in the number of sheep, Pajalić [32] reported that PS reached its lowest-ever numbers around 1950, with approximately 16,000 individuals. Historical information about the IS from this period is rather scarce and unreliable because the reports on the number of sheep in Istria made no distinction between the breeds (they were considered as a single population). According to genomic estimates, the steep N_e_ decline continued until about forty to fifty years ago (10–12 generations ago), and since then, N_e_ has remained fairly stable. According to the most recent N_e_ estimates, both breeds are not endangered, as N_e_ = 50 is recommended as the bottom line for endangered breeds [33]. However, due to substantially higher N_e_ in PS compared to IS, it can be concluded that PS has greater genetic variability and more potential to adapt to different environments and conditions.

As for inbreeding, the estimates were quite low in the population of PS (F_ROH>2_ = 0.029 and F_HOM_ = 0.039) and approximately twice as high in the population of IS (F_ROH>2_ = 0.062 and F_HOM_ = 0.058). Based on the previous pedigree-based analysis of IS (F_PED_ = 0.08, [34]) and PS (F_PED_ = 0.06, [31]), as well as on the previous genomic-based analysis of Drzaic et al. [10] on IS (F_ROH>2_ = 0.053, N = 25) and PS (F_ROH>2_ = 0.035, N = 40) we had some expectations on the inbreeding values in these populations. The results obtained generally agree with the latter study’s reports. Compared to the aforementioned study, this study better reflects the true levels of genomic inbreeding (F_ROH_) as it is based on a much larger and, thus, more representative sample. The F_ROH_ values reported for a couple of Italian non-selected local breeds ranged from 0.016 to 0.090 [11,35,36]; for Polish local breeds between 0.040 and 0.170 [37], and so on. Nowadays, there are many other reports on F_ROH_ for various sheep breeds worldwide and a frequent comparison of this parameter between different populations in many literature reports and scientific papers. Such comparisons are somewhat worthless considering the fact that F is a population-specific genetic parameter, and it will never have the potential for generalization. That is why we hereby advocate better contextualization and its usage in the best possible way in managing the available genetic resources in practice (e.g., to embed the estimates of F_ROH_ in genomic Optimum Contribution Selection). Since the results of ROH analysis are very sensitive to different input parameters for the detection of ROH segments (e.g., minimal number of SNPs in a ROH, maximal gap between neighboring SNPs, SNP density, etc.), to different methodology (consecutive vs. sliding window approach), to the number of genotyped animals, to SNP chip density, etc., comparisons between studies are practically impossible.

As for the F_ROH_ results for the different ROH length classes, there was evidence that both ancient and recent inbreeding affected these populations. However, since F_ROH>16_ made a predominant contribution to the overall F_ROH_, it can be concluded that recent inbreeding has mainly influenced the IS and PS genome. This conclusion was based on the fact that the coverage of the genome with short ROHs reflects ancestral inbreeding, while coverage of the genome with long ROHs reflects recent inbreeding [18]. Since both of the examined breeds suffer from recent inbreeding (contribution of F_ROH>16_ > 50% in both breeds), it is necessary to implement a well-designed mating plan that will minimize inbreeding and prevent the reduction of genetic diversity in the future. Limited financial resources for genotyping are certainly an obstacle to the implementation of this strategy in long-term routine genetic evaluation, so priority in the implementation of genomic OCS should definitely be given to IS. The rationale for this prioritization can be underpinned by the previously described 4-fold lower N_e_, and 2-fold higher F_ROH_ in IS compared to PS. The both determined parameters indicate more severe issues related to loss of genetic variability and increased risk of inbreeding depression in IS.

### 4.2. Selection Signatures and Functional Enrichment Analysis

In this comprehensive study, conducted on a relatively large number of genotyped animals per breed (N_IS_ = 1219 and N_PS_ = 2637), one genomic region with high level of autozygosity was detected and only present in IS. The specific ROH island was located at OAR6: 34–38 Mb containing several genes associated with milk production, growth and feed efficiency, immune response, and resistance (*SSP1*, *LCORL*, *HERC3*, *CCSER1*, *PKD2*, *PYURF* and *NAP1L5*). In the same genomic region and with the same analysis settings, Gorssen et al. [21] found ROH islands in 18 sheep breeds. However, only two breeds had complete overlapping of ROH island with IS (Merinizzata Italiana and Swiss White Alpine). In the study of Lukic et al. [38], which was conducted on eight local Croatian sheep breeds, there was also a signal of selection on OAR6 (32–40 Mb). But, their study considered the 8 breeds (including IS and PS) as one metapopulation (East Adriatic sheep breeds); and the signal was detected using different methodologies (integrated haplotype score (iHS), composite likelihood ratio test (CLR), extreme ROH islands (eROHi)) than the one implemented in this study.

In this study, the region on OAR6: 33.70–35.19 Mb corresponded to a genomic window harbouring *CCSER1*, which was reported to be related to economic traits in livestock species. It has been identified as a candidate gene for feed intake in 15 Russian sheep breeds [27] and marbling in Chinese Mongolian fat-tailed sheep [39]. In cattle, *CCSER1* has been identified as a candidate gene in selection signatures for beef cattle [40] and Jiaxian Red cattle [41] and was suggested to play a role in growth and feed efficiency. In addition, *CCSER1* has been associated with the number of spermatozoa in Assaf rams [42].

The region OAR6: 36.71–36.86 Mb was identified to contain *HERC3* that encodes a member of the HERC ubiquitin ligase family. Variation in this gene is associated with parasite resistance in Australian sheep [23]. Several studies have identified genes in relevant QTL regions on chromosome 6 that are associated with various traits in deferent cattle breeds. For example, Porto-Neto et al. [43] reported *HERC3* and Naserkheil et al. [44] reported 17 candidate genes of which *HERC3*, *NAP1L5*, *PYURF*, *PIGY*, *PKD2*, and *SPP1*, also found in the current study, were associated with yearling weight in cattle breeds. *HERC*, is also associated with milk protein percentage in Chinese Holstein populations [45]. Within an intronic region of *HERC3,* is located *NAP1L5* (https://www.ncbi.nlm.nih.gov/gene/101118611/; accesed on 15 April 2024). Highly expressed in eight different tissue types in adult cattle and in 15 different tissue types in fetuses, *NAP1L5* may play a role in growth and development [46].

The regions OAR6: 37.28–37.35 Mb and OAR6: 37.37–37.42 Mb were associated with *PKD2* and *SPP1*. Several scientific studies linked one or both genes with milk production and growth traits (mainly growth and yearling weight), such as Wei et al. [47] in Chinese local sheep breeds, Yurchenko et al. [27] in Russian local sheep breeds or Naserkheil et al. [44] in Korean cattle. Further, the role of *SPP1* and its association with the immune response to gastrointestinal parasites in Australian sheep were reported by Al Kalaldeh et al. [29]. Therefore, it can be concluded that *SPP1* is essential for vital biological processes, such as developmental processes, immunological responses, tissue and embryonic growth [48,49,50]. In addition, La et al. [28] reported that the amino acid sequence of SPP1 is highly conserved, i.e., the author reported a high percentage of sequence homology of sheep SPP1 with seven other species, ranging from 93% (homology with *Gallus gallus*) to 100% (homology with *Bos mutus*).

*LCORL* is one of the most frequently investigated gene affecting growth and conformation traits in human [51] and in animal species such as cattle [44], goats [52], pigs [53], and horses [54]. The hotspot on OAR6 also contained *LCORL*, which has been identified as a candidate gene in Chinese Merino sheep [55], and several sheep populations in France [56,57], and sheep populations in Switzerland [58]. *LCORL* has been associated with body weight in Australian Merino [59], growth and body weight in South African Mutton Merino sheep [60], and parasite resistance in Australian sheep [29]. Yurchenko et al. [27] reported candidate genes *LCORL* and *CCSER1* related to growth and feed intake in local sheep from Russia, while Matika et al. [61] and Zlobin et al. [62] reported an association between *LCORL* and muscle and fat deposition-related traits in sheep.

The ROH island OAR6:36.90–36.99 Mb also contained *PYURF*. Previous studies have shown that *PYURF* is a gene within a region that plays an important role in immunity underlying the resistance to gastrointestinal parasites in Australian sheep (OAR6: 34.7–39.2 Mb; [29]) as well as in Swedish cattle breeds, where *PYURF* gene is responsible for resistance to diseases and bacterial infections (BTA06: 37.64–37.72 Mb; [63]). A genome-wide association study in Korean Hanwoo cattle revealed selection signatures within the region BTA6: 38.37–38.99 Mb, which is comprised of 14 genes, including *PYURF* and *PIGY*, which are associated with meat traits. Furthermore, *PYURF* and *PIGY* genes were identified as candidate genes within the window of OAR6: 36.07–36.29 Mb, associated with clean fleece weight in Merino sheep from Uruguay [64].

Several genes from this study were associated with GO terms and KEGG pathways related to the regulation of: (1) biological characteristics (i.e., size, mass, shape, etc.); (2) homeostasis; (3) and response to environmental stimuli. Given the availability of SNP information, as well as the test-day dairy records (routine phenotyping in accordance with the ICAR standards), GWAS is our next step in order to uncover the genetic loci associated with the traits of selection interest. This will definitely provide us some additional important information helpful in the optimizing prediction of breeding values in these populations. However, more genotyped animals should be provided to empower the results of GWAS, but since additional genotyping and phenotyping of both breeds is still ongoing, enough large dataset for reliable GWAS will be probably available in the very near future.

## 5. Conclusions

The aim of this study was to estimate the effective population size (N_e_), inbreeding and signatures of selection in Istrian (IS) and Pag sheep (PS). The results obtained provide an insight into the current status of the populations studied at the genomic level and form a good basis for setting up the future selection strategy (mating plans) for IS and PS. The estimated N_e_ (IS = 197, PS = 838), and F_ROH>2_ (IS = 6.24, PS = 2.88) both indicate larger issues related to loss of genetic variability and increased risk of inbreeding depression in IS compared to PS. A substantial portion of F_ROH>8_ Mb (and especially F_ROH>16_ Mb) in the overall F_ROH_, indicate a high proportion of recent inbreeding in both breeds suggesting that more efforts should be made to keep inbreeding as low as possible to prevent future genetic erosion in these populations. Implementing the basic principles of genomic OCS, could be a solution for increasing genetic gain while controlling the rate of inbreeding and maintaining genetic diversity in these local breeds. Based on the findings of this study and considering limited financial resources for genotyping, priority in the implementation of genomic OCS should be given to IS. Regarding the identified ROH islands harboring genes associated with economically important traits, further research is needed to exploit this information for genetic improvement of traits important to IS and PS.

## Figures and Tables

**Table 1:** Summary of the runs of homozygosity (ROH) results per breed: number of ROH segments per animal, number and corresponding proportion of ROH classes and genomic inbreeding coefficients F_HOM_ and F_ROH>2_. The segment sizes of ROH and F_ROH_ are calculated according to four different classes 2–4 Mb, 4–8 Mb, 8–16 Mb, and >16 Mb.

		Istrian Sheep (IS)	Pag Sheep (PS)
nROH/animal		13.05	5.94
ROH classes	ROH_2–4_ ROH_4–8_ ROH_8–16_ ROH_>16_	2320 (15%) 6683 (43%) 4067 (26%) 2506 (16%)	2961 (19%) 6147 (39%) 3779 (24%) 2767 (18%)
Inbreeding coefficients /classes (%)	F_HOM_	5.80	3.90
	F_ROH>2_	6.24	2.88
	F_ROH2–4_	0.26	0.22
	F_ROH4–8_	1.65	0.61
	F_ROH8–16_	2.14	0.75
	F_ROH>16_	3.72	1.58

**Table 2:** Details of runs of homozygosity (ROH) hotspots detected in Istrian sheep.

Chr	Start (bp)	End (bp)	Length (bp)	Gene	Gene_ID	Description	Functions
6	33,699,466	35,188,219	1,488,754	CCSER1	ENSOARG 00000007488	Coiled-coil serine rich protein 1	Growth, feed intake, reproduction
6	36,709,616	36,855,827	146,212	HERC3	ENSOARG 00020023461	HECT and RLD domain containing E3 ubiquitin protein ligase 3	Growth, immune responses
6	36,739,315	36,741,723	2409	NAP1L5	ENSOARG 00020037274	Nucleosome assembly protein 1 like 5	Growth and development (chromatin organization)
6	36,902,696	36,990,169	87,474	PYURF	ENSOARG 00020035580	PIGY-phosphatidylinositol glycan anchor biosynthesis class Y	Growth, immune response
6	37,282,161	37,348,326	66,166	PKD2	ENSOARG 00020024032	Polycystin 2, transient receptor potential cation channel	Growth, milk, feed intake
6	37,369,583	37,425,206	55,624	SPP1	ENSOARG 00020024091	Secreted phosphoprotein 1 (osteopontin)	Growth, milk, immune responses
6	38,052,622	38,222,929	170,308	LCORL	ENSOARG 00020024486	Ligand dependent nuclear receptor corepressor like	Growth

## Data Availability

The data that support the findings of this study are available on request from the corresponding author, J.R. The data are not publicly available due to legislation and ownership details, as the Association of Sheep and Goat Breeders in the Republic of Croatia and CAAF provided a significant share of the samples.

## Supplementary Materials

The following supporting information can be downloaded at: https://www.mdpi.com/article/10.3390/ani14131928/s1, Table S1: Information about sampling of Istrian sheep (IS) and Pag sheep (PS) before pruning; Table S2: QTL enrichment results for the Istrian sheep (IS); Table S3: Functional annotation of five genes in Istrian sheep (IS).
